# Muscle and tendon adaptations to moderate load eccentric vs. concentric resistance exercise in young and older males

**DOI:** 10.1007/s11357-021-00396-0

**Published:** 2021-07-01

**Authors:** Jonathan Iain Quinlan, Martino Vladimiro Franchi, Nima Gharahdaghi, Francesca Badiali, Susan Francis, Andrew Hale, Bethan Eileen Phillips, Nathaniel Szewczyk, Paul Leonard Greenhaff, Kenneth Smith, Constantinos Maganaris, Phillip James Atherton, Marco Vincenzo Narici

**Affiliations:** 1 School of Sport, Exercise and Rehabilitation Sciences, University of Birmingham, Birmingham, UK; 2 National Institute for Health Research, Birmingham Biomedical Research Centre At University Hospitals Birmingham NHS Foundation Trust, Birmingham, UK; 3 MRC Versus Arthritis Centre for Musculoskeletal Ageing Research and NIHR Nottingham Biomedical Research Centre, University of Nottingham’s Royal Derby Hospital Centre, Nottingham, UK; 4 Department of Biomedical Sciences, University of Padova, Padova, Italy; 5 Sir Peter Mansfield Magnetic Resonance Centre, School of Physics and Astronomy, University of Nottingham, Nottingham, UK; 6 Ohio Musculoskeletal and Neurological Institute (OMNI) and Department of Biomedical Sciences, Ohio University, Athens, OH 43147 USA; 7 School of Sport and Exercise Sciences, Liverpool John Moores University, Liverpool, UK; 8 CIR-MYO Myology Center, University of Padova, Padova, Italy

**Keywords:** Tendon, Eccentric, Muscle, Submaximal RET, Ageing

## Abstract

Resistance exercise training (RET) is well-known to counteract negative age-related changes in both muscle and tendon tissue. Traditional RET consists of both concentric (CON) and eccentric (ECC) contractions; nevertheless, isolated ECC contractions are metabolically less demanding and, thus, may be more suitable for older populations. However, whether submaximal (60% 1RM) CON or ECC contractions differ in their effectiveness is relatively unknown. Further, whether the time course of muscle and tendon adaptations differs to the above is also unknown. Therefore, this study aimed to establish the time course of muscle and tendon adaptations to submaximal CON and ECC RET. Twenty healthy young (24.5 ± 5.1 years) and 17 older males (68.1 ± 2.4 years) were randomly allocated to either isolated CON or ECC RET which took place 3/week for 8 weeks. Tendon biomechanical properties, muscle architecture and maximal voluntary contraction were assessed every 2 weeks and quadriceps muscle volume every 4 weeks. Positive changes in tendon Young’s modulus were observed after 4 weeks in all groups after which adaptations in young males plateaued but continued to increase in older males, suggesting a dampened rate of adaptation with age. However, both CON and ECC resulted in similar overall changes in tendon Young’s modulus, in all groups. Muscle hypertrophy and strength increases were similar between CON and ECC in all groups. However, pennation angle increases were greater in CON, and fascicle length changes were greater in ECC. Notably, muscle and tendon adaptations appeared to occur in synergy, presumably to maintain the efficacy of the muscle–tendon unit.

## Introduction

Ageing affects both the skeletal muscle and tendon, leading to quantitative as well as qualitative tissue alterations, impacting on muscle function and mobility [50]. The loss of muscle mass and strength, known as sarcopenia, has a complex aetiology involving neurodegenerative processes, changes in protein metabolism, oxidative stress, inflammation and chronic inactivity [12, 13, 45]. Quantitatively, approximately 30% of muscle mass is lost from 20 to 80 years of age [31]. However, the associated loss of muscle strength is approximately fourfold greater than that of muscle size, pointing to a significant deterioration of muscle quality (loss of force per unit of muscle cross-sectional area) with increasing age [48]. Decreased muscle activation [29], progressive muscle fibre denervation [70], neuromuscular junction damage [27], reduced excitation–contraction coupling [15] and reduced single fibre-specific tension [14] are likely to collectively contribute to this loss of muscle quality. However, muscles in vivo exert their forces through tendons, and hence, when considering muscle function and its determinants, it is important to consider the muscle–tendon complex rather than the muscle alone. This is because the mechanical behaviour of the muscle–tendon unit (MTU) is not only affected by the mechanical characteristics of the muscle itself, but also by the elastic properties of the in-series tendon [55, 62]. In order for a muscle to generate maximal force, muscle fibres must operate close to the region of optimal myofilament overlap during muscle contraction. Muscles shorten during an isometric contraction, and the degree of shortening in muscle fibres is affected by tendon stiffness [41],thus, changes in stiffness are expected to impact on muscle fibre shortening and thus on the operating length range of the muscle. Notably, tendon stiffness and Young’s modulus have both been shown to be reduced in older individuals [53, 55, 71, 72], although it has also been shown that no changes occur with ageing [6, 10]. Irrespectively, both muscle and tendon tissues retain considerable plasticity in response to chronic loading in old age since sarcopenia and tendon stiffness can be substantially improved by high-intensity resistance exercise training (RET) (≥ 70% 1RM) [16, 52, 59, 69]. It has also been shown that moderate-intensity RET (60% 1RM) can lead to improvements in muscle mass and function in older individuals [73]. Such moderate mechanical loads may offer a practical alternative to high-intensity RET for older males (OM), with a lower metabolic demand and a reduced injury risk. However, whether this submaximal loading would be sufficient to elicit positive changes in tendon biomechanical properties is unknown. Indeed, tendon adaptation has been shown to be dependent upon the amount of strain [1, 38] and hence training load. As such, low training loads (~ 40% 1RM) have been suggested to be insufficient to induce positive changes to tendon biomechanical properties [28],even when controlled for overall work [34], reinforcing the concept that the magnitude of acute loading may be key for tendon adaptation.

Most conventional RET programmes involve consecutive concentric (CON) and eccentric (ECC) contractions. ECC contractions are known to produce significantly greater force than CON and even isometric contractions [22, 33], enabling the use of heavier loads in ECC than in CON contractions [65]. Furthermore, it is known that ECC contractions can perform the same absolute muscle work of CON contractions but at a lower metabolic cost [3]. Thus, it is not surprising that ECC RET represents an attractive form of training for the elderly, particularly when ECC strength seems to be maintained to a great extent than CON strength in OM [64]. Nonetheless, whilst many studies have compared the muscle adaptations to CON and ECC RET in young males (YM), data on OM are scanty. Previous research has shown that in YM, there is no significant difference between CON and ECC in terms of overall hypertrophy or strength gain, when matched for either work or maximum relative load [21, 22]. Similarly, no difference in the time course of muscle adaptation existed between CON only leg and CON + ECC RET, despite greater mechanical load in ECC + CON,albeit, this study did not compare isolated CON and ECC contractions [43]. However, two distinct modes of muscle architectural remodelling have been found in response to CON and ECC training. CON RET results in a greater increase in fascicle pennation angle compared to ECC, and ECC leads to a greater increase in fascicle length compared to CON [21, 57]. Thus, these opposing adaptations suggest that the placement of new contractile material in response to chronic overloading may differ between CON and ECC RET, such that CON RET may result in a greater addition of sarcomeres in parallel and ECC RET may result in a greater addition of sarcomeres in series [24].

As mentioned above, ECC contractions allow for the use of greater training loads when compared to CON or conventional RET. As such, it would be logical to suggest that these greater training loads and hence greater strain on the corresponding tendon may lead to greater or faster tendon adaptation compared to CON or conventional RET. It is perhaps for this reason that ECC protocols are often utilised for tendinopathies [11, 25, 32]. Unfortunately, less is known about the adaptability of healthy human tendon to ECC loading [56]. It has been shown that the patellar tendon (PT) of YM adapts to high-intensity ECC RET (80% 1RM) by increasing both tendon stiffness and Young’s modulus [42]. Significantly, the change observed did not differ to CON RET group [42], despite higher training loads in the ECC group. However, the comparison of CON and ECC RET for tendon adaptation has not been investigated in an older population nor has the comparison of YM and OM PT adaptation to CON or ECC RET. Therefore, it currently remains unknown whether there are any differences in tendon adaptation to CON or ECC RET in an older population or whether these adaptations differ to a younger cohort. Indeed, the adaptations to each training modality may differ in either the magnitude or the time course. However, in order to answer the second portion of this, a more complete time course of PT adaptation must first be identified. Unfortunately, the overwhelming majority of studies on tendon adaptability only consider pre- and post-intervention time points and thus may have missed crucial information on the rate of adaptability. This temporal information has key implications for the design and implementation of training interventions aimed at increasing muscle mass and strength. Indeed, one would expect the progressive increase in muscle strength produced by overloading to be matched by an increase in tendon stiffness to enable the tendon to withstand the large forces generated by the muscle against the training load. In principle, this increase in stiffness may be achieved either by increasing the tendon cross-sectional area or by improving tendon material properties. However, whilst it is fundamental for these tendon adaptations to occur in a coordinated fashion with those of muscle function, it is not known if they precede, occur pari passu, or follow the increase in strength. Hence, knowledge of the time course of the muscular and tendinous adaptations seems essential for understanding the mechanisms responsible for the improvement of muscle function obtained with chronic overloading.

Therefore, the aim of this study was threefold: (1) to investigate possible differences between the tendon adaptations of young and old individuals to CON and ECC RET, (2) to ascertain if contraction-specific muscle adaptations to CON and ECC RET occur with moderate loads and (3) to compare the time course of MTU adaptation in response to moderate load RET for young and older populations.

## Methods

### Subject recruitment and ethics

A total of 37 healthy, recreationally active individuals were recruited, of which 20 were young males (YM) (18–35 years) and 17 were older males (OM) (65–73 years). These individuals were randomly assigned to either CON or ECC only training creating the following four groups: Young concentric (YC, *n* = 10), young eccentric (YE, *n* = 10), old concentric (OC, *n* = 8) and old eccentric (OE, *n* = 9). Both YM groups and both OM groups were matched for age, height, body mass or body mass index (BMI) (Table 1). All participants underwent full medical screening prior to enrolment, whereby those with any musculoskeletal, metabolic, respiratory, neurological or cardiovascular medical conditions were excluded from the study. Potential participants were excluded from the study if they took part in regular resistance exercise. All participants provided informed written consent to this study, which was approved by the University of Nottingham Ethics Committee (ethics approval, B13032014 SoMSGEM) and complied with the Declaration of Helsinki.

**Table 1:** Participant characteristics for all groups

	Age (yrs)	Height (cm)	Weight (kg)	BMI (kg/m^2^)
Young CON	23.3 (3.8)	175 (1.3)	74.2 (4.4)	24.1 (3.8)
Young ECC	25.3 (6.2)	176 (2.2)	73 (6.1)	23.3 (3.9)
Old CON	69.1 (3.0)	175 (3.3)	79.2 (8.5)	25.7 (2.2)
Old ECC	67.5 (1.5)	176 (5.8)	76.8 (10.4)	24.7 (2.9)

Values are presented as mean (± SD). *N* values for groups were as follows: YC, *n* = 10; YE, *n* = 10; OC, *n* = 8; and OE, *n* = 9

### Resistance exercise training

RET was performed using a specialised leg-press machine (Technogym, Gambettola, Italy), adapted to exclusively allow either ECC or CON contractions, a setup that has previously been used and described in detail [21, 24]. Participants were randomly assigned to either CON only or ECC only RET and completed 8 weeks of RET, performed with a load intensity of either 60% of CON or 60% ECC 1RM, depending upon their assignment. The training was performed 3 times per week for 8 consecutive weeks, with each session consisting of 4 sets of 15 repetitions. For each mode of contraction, each repetition lasted 3 s, and there was a 2-min rest period between sets. The initial 4 weeks of training were bilateral in nature,however, from week 5 onwards, the training was altered to unilateral RET. This occurred as at the end of week 4, PT biopsies were performed in the non-dominant limb (separate analysis), which necessitated avoiding training the leg of the biopsied tendon for the last 4 weeks of the study to avoid potential injury. For that time period (i.e. week 5 onwards), training load was quantified via 60% of a unilateral 1RM assessment. All testing and imaging were carried out upon the dominant limb, which trained throughout the full 8-week period.

As the aim of this study was to investigate the effects of CON and ECC, it was essential to ensure similar levels of neural activation within each contraction mode, a fundamental requirement of the force–velocity relationship [8]. Thus, we ensured that for the subjects in the ECC group, the neural drive during an ECC 10RM was equal to that of a CON 10RM. Matching for activation was carried out in a similar fashion to previous work [21, 24]. Briefly, the integrated EMG activity of the vastus lateralis (VL) was calculated over a 200 ms time frame during the mid-portion of the contraction phase for a total of 5 contractions, whereby the average was utilised for comparison between CON and ECC. The contractions were deemed neurally matched when the average values were within 10% of one another, as based on previous work [21, 24].

### Maximum voluntary contraction (MVC)

Maximal quadriceps isometric muscle torque (i.e. muscle strength) was measured at weeks 0, 2, 4, 6 and 8 using an isokinetic dynamometer (Cybex Norm, Cybex International Inc., NY, USA) over 3 joint angles, 60°, 70° and 80°, with full extension corresponding to 0°. After a brief warm-up, subjects performed two MVCs at each angle, which lasted for 4 s, with a rest period of 30 s between the 2 contractions and 2 min between joint angles. Subjects were provided with real-time visual feedback of torque production during each isometric contraction. The maximum isometric torque value obtained over the three joint angles was chosen as the MVC peak value. The maximum isometric torque value produced at each joint angle was taken in order to analyse the time course of changes in the knee extensors angle–torque relationship. The optimal angle was taken as the joint angle at which the peak of the maximum isometric torque was produced.

### Electromyography (EMG)

EMG was acquired from the belly of the VL and the biceps femoris muscles of the dominant limb using surface EMG electrodes to estimate muscle activation. EMG was utilised during the assessment of tendon biomechanical properties and muscle activation capacity (explained in detail later). The skin was thoroughly prepared through the removal of hair, light abrasion and cleaned with 0.1% w/w alcohol wipes. Raw EMG signals were sampled at 2.0 kHz and digitised with an analogue to digital converter (BioPac MP150, BIOPAC Systems Inc.) and filtered and amplified. The root mean square was calculated through an Acqknowledge software function (Acqknowledge 4.2.0, BIOPAC systems Inc.) [21].

### Muscle architecture

Muscle architecture provides key information in regard to the structure of the muscle as well providing information on function. Measures including VL fascicle length and pennation angle were assessed at weeks 0, 2, 4, 6 and 8 via the acquisition and analysis of images using B-mode ultrasonography (Mylab 70, Esaote Biomedica) with a 100-mm, 10–15 MHz, linear array probe. Images were obtained whilst the participant was at rest and lying supine upon a bed, in the same manner as presented in previous work [24]. Briefly, images were acquired at 50% of the VL length and midsagittal line of the muscle. The transducer was aligned to the fascicle plane allowing optimal capture of the fascicles [21, 57]. Data were collected and analysed by the same operator to remove any inter-operator error. Digital analysis of the images was completed using ImageJ (ImageJ 1.52a). Manual linear extrapolation of fascicle length values was only adopted in case a fascicle was not visible in its entirety: because of the large field of view (100 mm), on average, 2 to 3 fascicles per image were fully digitised, whereas the rest of the analysed fascicles (5 per image, assessed in 3 different images) were subjected to minimal manual linear extrapolation, practically minimising the effect of extrapolation pitfalls for muscle architecture analyses [20]. Data was omitted for one subject in the YE group (*n* = 9) as the full-time course was not obtained due to technical difficulties at one time point prevented accurate analysis.

### Quadriceps muscle volume

In order to measure muscle hypertrophy, a 3 T MRI scanner was utilised to obtain quadriceps anatomical cross-sectional area (ACSA) and consequently total quadriceps muscle volume. Transversal scans were performed with participants lying supine, with the knee joint fixed at 180°. Quadriceps ACSA was manually measured using digital analysis software (OsiriX Lite 9.5.2, Pixmeo SARL). Consequently, whole quadriceps volume was obtained utilising the previously proven method [60] of the truncated cone formula, shown below. Quadriceps ACSA was assessed every 7 slices whereby slice thickness was set at 3 mm, with no interslice gap (i.e. 21-mm intervals). An average total of 21 slices were used to digitise quadriceps volume. Quadriceps muscle volume was calculated at week 0, week 4 and week 8. Data were analysed by the same operator to remove any inter-operator error during the digitisation process. One participant in the OE group was unable to have any of the MRI scan due to medical reasons (OE, *n* = 8):

$$V=\frac{1}{3}\bullet d [a+\sqrt{(a\bullet b)}+b]$$

*Truncated cone formula* *:* *V, volume; d, distance between slices; a, first ACSA value; b, second ACSA value*

### Patellar tendon cross-sectional area

In order to measure tendon size, PT cross-sectional area (CSA) was obtained via two previously validated techniques. For weeks 0, 4 and 8, a 3 T MRI scanner was utilised, and these images were obtained in the same participant setup as described above. PT CSA images were consequently manually measured via digital analysis software (OsiriX Lite 9.5.2, Pixmeo SARL), whereby an average value was generated for CSA along the length of the PT, similar to previous work [55, 69]. However, due to logistical challenges, it was not possible to carry out MRI scanning at weeks 2 and 6,therefore, B-mode ultrasonography with a 50-mm, 10–15 MHz, linear array probe was utilised to obtain PT CSA at weeks 2 and 6. Scans were obtained every 1 cm along the full length of the tendon in order to consider regional differences. Consequently, images were manually measured via ImageJ (ImageJ 1.52a), and the average of these scans generated a value for PT CSA. In order to confirm that the interchangeable use of both techniques would not influence our results, 8 random participants (2 from each group) were selected for further analysis. PT CSA values were consequently calculated at week 0 assessments via both ultrasound and MRI analysis for all 8 participants, and a Pearson’s correlation test was carried out via GraphPad Prism Software, version 8 (La Jolla, CA, USA).

### Tendon biomechanical properties

Due to quality issues with some video files (i.e. poor clarity of both origin and insertion points throughout the full clip), data was not available for some participants at all time points. Therefore, the numbers included for analysis of tendon material properties at weeks 0, 2, 4, 6 and 8 were as follows: YC, *n* = 10; YE, *n* = 9 (i.e. -1); OC, *n* = 8; and OE, *n* = 8 (i.e. -1). Measures of tendon biomechanical properties describe tendon function and integrity (including PT stiffness and Young’s modulus). These measures were obtained in similar fashion to the groups’ previous work [42, 55, 69]. PT length (L0) and tendon elongation were obtained by sagittal-plane real-time B-mode ultrasonography with a 10-cm linear array probe. L0 was defined as the distance between the apex of the patella and the tuberosity of the tibia at rest at 90° knee joint angle. Tendon elongation was measured as the distance between the above anatomical landmarks during a voluntary isometric ramped contraction (at 90° knee joint angle) over a 5-s period. The measurement of elongation was obtained by a piece of semi-automated pixel tracking software (Tracker v4.95, OpenPhysics), which was run in triplicate, and the average pixel movement was used for further analysis. Moreover, to prevent an overestimation of tendon elongation due to translation of the tibia, a calibrated goniometer was attached to the lateral side of the tested knee. In order to calculate PT stiffness, true knee extensor torque was calculated as the sum of the measured knee extension torque and the antagonistic torque, estimated through the root mean square of the EMG acquired from the biceps femoris [28, 54]. Consequently, the PT force was calculated by dividing the true knee extensor torque by the estimated PT moment arm [74]. Force–elongation data were fitted with a second-order polynomial curve, which allowed the assessment of PT stiffness, defined as the gradient of the force–elongation curve over the final 10% of maximal force [55, 58]. Young’s modulus was calculated as tendon stiffness multiplied by the ratio of tendon length over tendon CSA.

### Statistical analysis

All data herein are presented as mean ± SD. Differences within group were analysed via repeated-measures two-way ANOVA with a Bonferroni correction using GraphPad Prism software, version 8 (La Jolla, CA, USA). Differences between group (effect of age and contraction type) were also assessed with the above method. The level of significance was set at *P* < 0.05.

## Results

### Tendon biomechanical properties

**Fig. 1:**
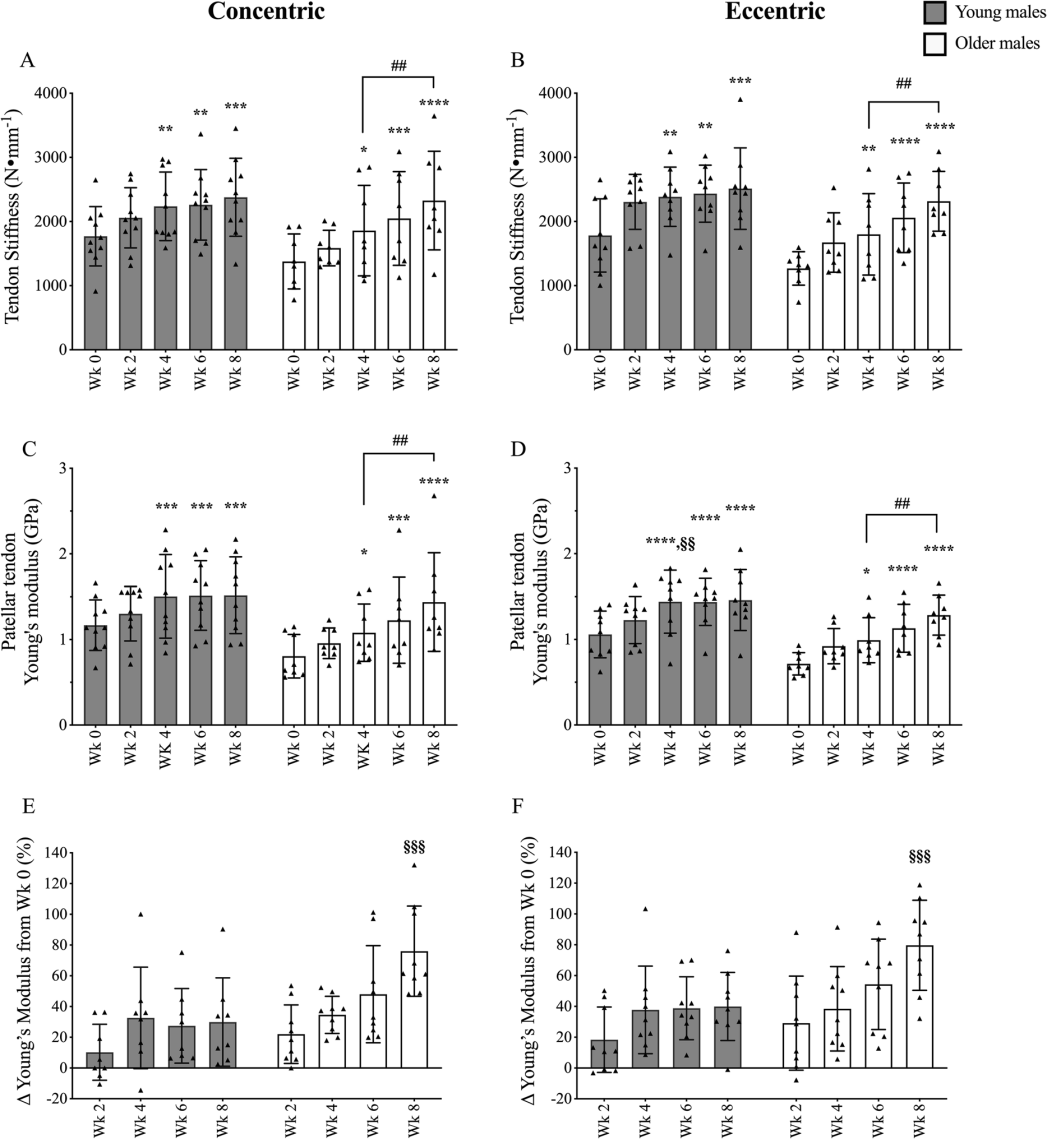
Time course of the patellar tendon biomechanical adaptation in response to submaximal CON (**A**, **C**, **E**) or ECC RET (**B**, **D**, **F**). N values for groups were as follows: YC, *n* = 10; YE, *n* = 9; OC, *n* = 8; and OE, *n* = 8. *P* values for time point vs baseline in respective group * *P* < 0.05, ** *P* < 0.01, *** *P* < 0.001, **** *P* < 0.0001. P values for week 4 vs week 8 in respective group, ## *P* < 0.01. P values for young vs old at respective time point §§ *P* < 0.01, §§§ *P* < 0.001

Firstly, the comparison of ultrasound and MRI for the measurement of tendon CSA showed good agreement between the two techniques (r^2^ = 0.99, *p* < 0.0001, *n* = 8, Y = 0.847*X + 14.30). The slope generated suggests that US typically underestimates tendon CSA in comparison to MRI, which appears evident in all groups at time points 2 and 6 (i.e. when US was utilised) (Table 2). There was no observed change to either PT length (L0) or PT CSA at any time point in any group (Tables 2 and 3). Within YM, PT stiffness and Young’s modulus were significantly increased in both CON and ECC by week 4 and were maintained until week 8, with no additional increase from weeks 4 to 8. Further, there was no difference between the PT stiffness and Young’s modulus at any time point nor percentage change in Young’s modulus of YC and YE (Fig. 1). Whilst PT stiffness and Young’s modulus were significantly increased by week 4 and maintained to week 8 in both YC and YE, in OM, there was also a significant increase from weeks 4 to 8 (Fig. 1). Similar to YM, there was no significant difference between the values obtained for OC and OE in OM. There was no difference between the baseline values of any group. However, the percentage change in Young’s modulus at week 8 was significantly higher in both the OM groups compared to the YM group (i.e. YC vs OC and YE vs OE, Fig. 1).

**Table 2:** Mean (± SD) values for patellar tendon CSA (mm^2^) throughout the training period

	Week 0	Week 2	Week 4	Week 6	Week 8
Young CON	84.2 (5.6)	83.4 (5.8)	84.8 (5.3)	83.3 (5.7)	85.1 (5.0)
Young ECC	86.9 (10.2)	86.6 (10.2)	86.8 (10.7)	86.3 (9.9)	86.6 (10.8)
Old CON	89.0 (11.1)	88.3 (10.7)	89.0 (11.3)	88.4 (10.7)	89.1 (11.5)
Old ECC	89.7 (10.4)	89.3 (11.0)	89.6 (10.7)	90.0 (10.0)	90.5 (9.3)

*N* values for groups were as follows: YC, *n* = 10; YE, *n* = 9; OC, *n* = 8; and OE, *n* = 8

**Table 3:** Mean (± SD) values for patellar tendon length (mm) throughout the training period

	Week 0	Week 2	Week 4	Week 6	Week 8
Young CON	54.5 (8.0)	53.7 (8.1)	54.7 (8.5)	54.8 (7.8)	53.7 (8.9)
Young ECC	49.4 (5.3)	48.4 (5.2)	48.9 (4.9)	49.4 (4.2)	49.7 (3.4)
Old CON	51.0 (4.0)	51.7 (3.7)	50.9 (3.7)	51.6 (5.0)	51.6 (5.3)
Old ECC	53.4 (2.6)	52.8 (4.2)	52.3 (2.2)	52.8 (2.1)	53.1 (3.0)

*N* values for groups were as follows: YC, *n* = 10; YE, *n* = 9; OC, *n *= 8; and OE, *n* = 8

### Muscle morphology and architecture

**Fig. 2:**
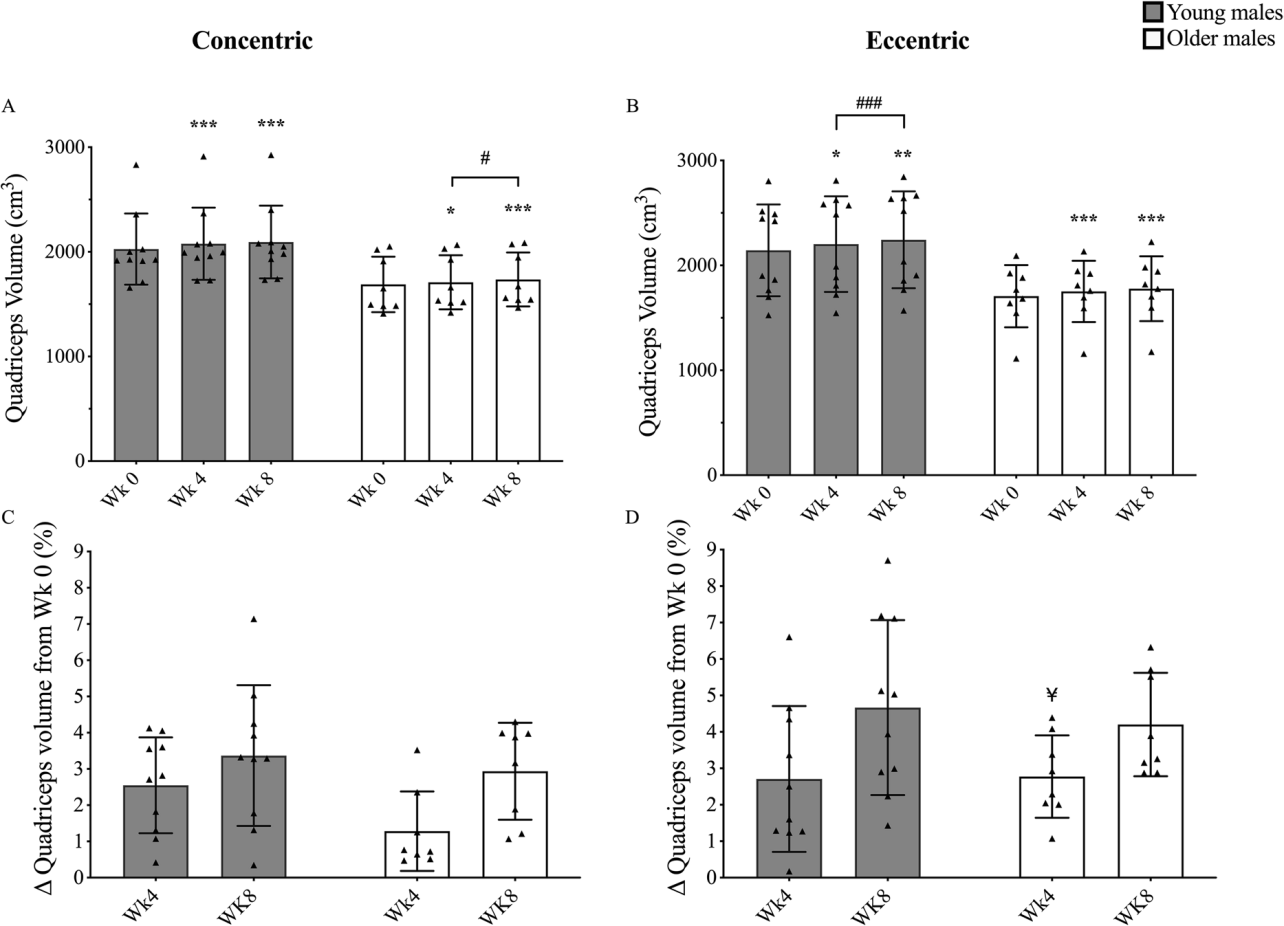
Time course of quadriceps’ muscle volume adaptation in response to submaximal CON (**A + C**) or ECC (**B + D**) RET. *N* values for groups were as follows: YC, *n* = 10; YE, *n* = 10; OC, *n* = 8; and OE, *n* = 8. P values for time point vs baseline in respective group * *P* < 0.05, ** *P* < 0.01, *** *P* < 0.001. P values for week 4 vs week 8 in respective group, # *P* < 0.05, ## *P* < 0.01. *P* values for CON vs ECC in respective age group and time point ¥ *P* < 0.05

**Fig. 3:**
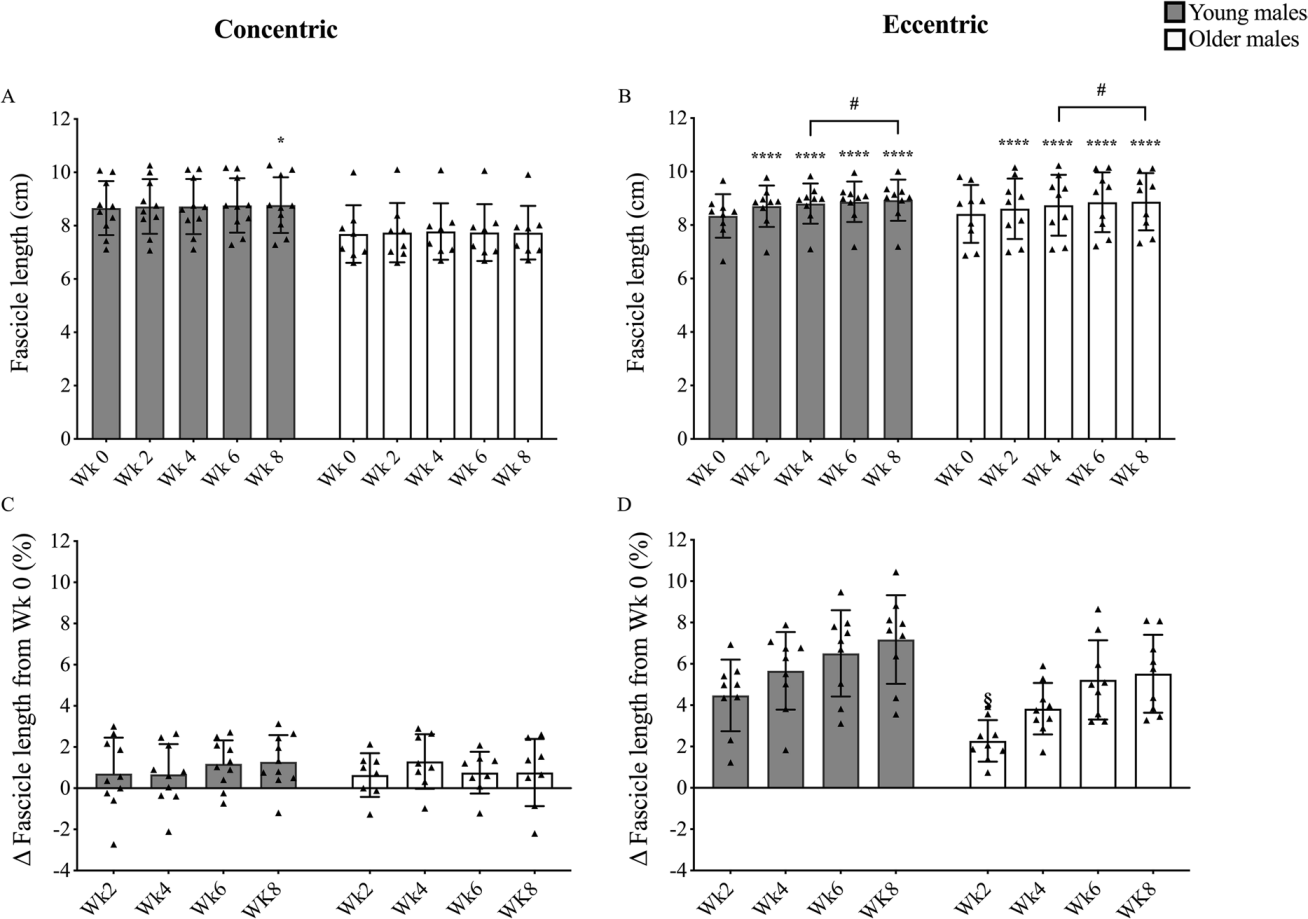
Time course of the vastus lateralis fascicle length adaptation in response to submaximal CON (**A + C**) or ECC (**B + D**) RET. *N* values for groups were as follows: YC, *n* = 10; YE, *n* = 9; OC, *n* = 8; and OE, *n *= 9. P values for time point vs baseline in respective group * *P* < 0.05, **** *P* < 0.0001. *P* values for week 4 vs week 8 in respective group, # *P* < 0.05. *P* values for young vs old at respective time point § *P* < 0.05

**Fig. 4:**
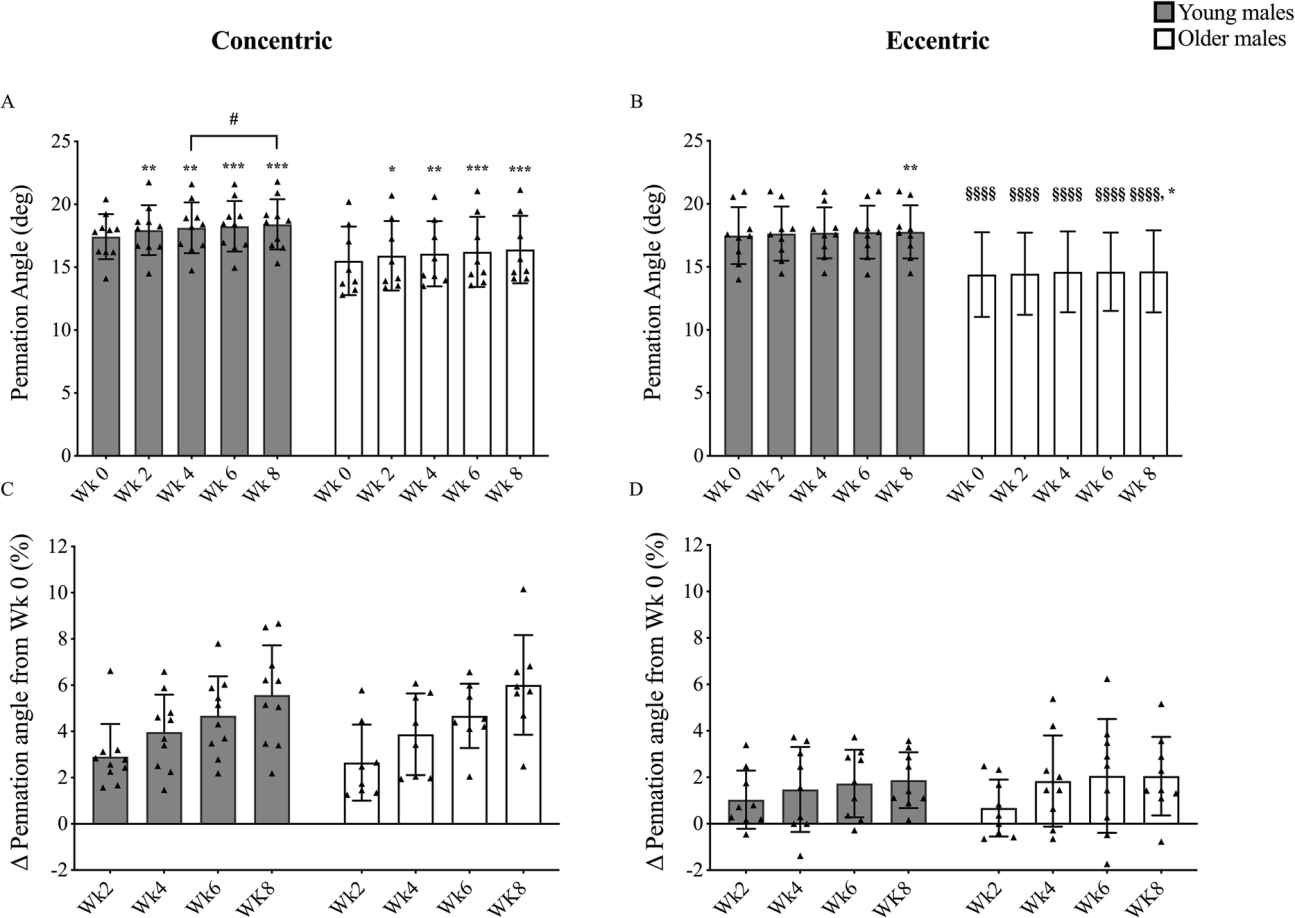
Time course of the vastus lateralis pennation angle adaptation in response to submaximal CON (**A + C**) or ECC (**B + D**) RET. *N* values for groups were as follows: YC, *n* = 10; YE, *n* = 9; OC, *n* = 8; and OE, *n* = 9. *P* values for time point vs baseline in respective group * *P* < 0.05, ** *P* < 0.01, *** *P* < 0.001. *P* values for week 4 vs week 8 in respective group, # *P* < 0.05. *P* values for young vs old at respective time point §§§§ *P *< 0.0001

A significant increase in quadriceps muscle volume was observed at every time point, in each group and in response to both training modes (Fig. 2). The OE group showed a significantly greater response in the % increase in volume when compared to the age-matched OC group (2.8 ± 1.1% vs. 1.3 ± 1.1%, *P* < 0.05, respectively). Increases in Lf were found for both young (from week 2) and older males (from week 4) but only after eccentric training (Fig. 3). Conversely, greater changes in PA were found already after 2 weeks of concentric training for both YM and OM (Fig. 4). However, PA significantly increased in the YE group at week 8 and in the OE group at week 4 and thereafter, although these changes were modest compared to YC and OC groups (Fig. 4). For any given training modality, the magnitude of changes in muscle architecture parameters was higher in the younger group compared to the older group.

### Muscle functional properties

**Fig. 5:**
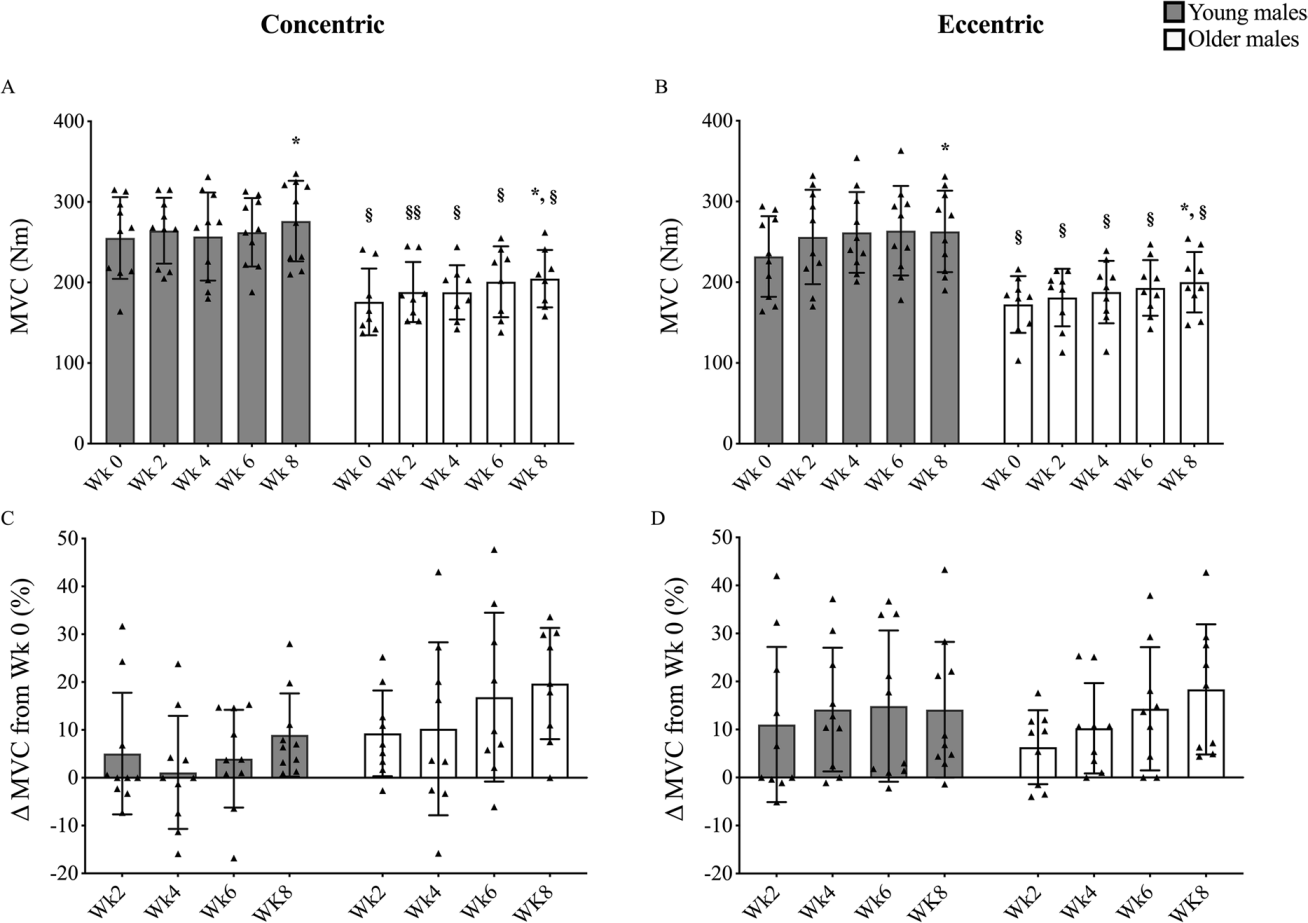
Time course of knee extension MVC_peak_ adaptation in response to submaximal CON (**A + C**) or ECC (**B + D**) RET. *N* values for groups were as follows: YC, *n* = 10; YE, *n* = 10; OC, *n* = 8; and OE, *n* = 9. *P* values for time point vs baseline in respective group * *P* < 0.05. *P* values for *P* values for young vs old at respective time point § *P* < 0.05, §§ *P* < 0.01

Maximum isometric voluntary torque presented a trend for increase in all groups at all time points and reached significance only at week 8 (Fig. 5). Furthermore, training load (Table 4) significantly increased at week 4 vs. baseline for both YM and OM (*p* < 0.05 and *P* < 0.01, respectively, bilateral training mode) and at week 8 vs. week 4 (*P* < 0.05 and *P* < 0.01, respectively, unilateral training mode).

**Table 4:** Mean (± SD) values for the progressive training load (kg) used by each RET group throughout the training period

	Week 0–2	Week 2–4	Week 4–6	Week 6–8
Young CON	184.8 (41.7)	202.7 (46.9) *	112.5 (29.1)	128.3 (28.8) *
Old CON	133.2 (26.9) §	151.3 (29.9) **,§	75.8 (5.9) §	90.8 (8.6) **,§
Young ECC	261.3 (41.5)	278.6 (39.7) *	146.9 (18.7)	166.9 (17.9) *
Old ECC	188.3 (48.9) §§	207.1 (51.9) **,§§	118.2 (32.6) §	135 (32.7) ***,§

sl, single leg training load value. *N* values for groups were as follows: YC, *n* = 10; YE, *n* = 10; OC, *n* = 8; and OE, *n* = 9. *P* values for time point vs either 0–2 weeks value (for 2–4 weeks) or 4–6 sl value (for 6–8 sl weeks value) in respective group * *P* < 0.05, ** *P* < 0.01, *** *P* < 0.001. *P* values for young vs old at respective time point and between respective groups § *P* < 0.05, §§ *P* < 0.01

**Fig. 6:**
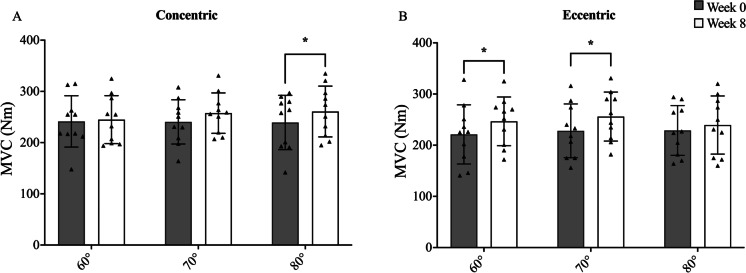
The angle–torque relationship in YC (**A**) and YE (**B**) at week 0 and week 8. *N* values for groups were as follows: YC, *n* = 10; YE, *n* = 9. P values between week 0 and week 8 in respective group * *P* < 0.05

The angle at which the MVC_peak_ was achieved showed a significant shift towards knee flexion after CON (+ 8.9°, being 0 = leg fully extended, *P* < 0.01) and a significant shift towards knee extension after ECC training (-7°, *P* < 0.01) in YM (Fig. 6). Thus, in YE, the torque produced at distinct joint angles showed the following changes after 8 weeks: 60° =  + 14.4%, *P* < 0.05, and 70° =  + 14.3%, *P* < 0.05; 80° =  + 5%, N.S., full knee extension = 0°. Conversely, YC presented almost opposite adaptations 60° =  + 2.6%, N.S.; 70° =  + 8.1%, N.S.; 80° =  + 10.7%, *P* < 0.05). Similar shifts were observed in the OE group, but these differences did not reach statistical significance.

## Discussion

Our study sought to investigate the effects of submaximal CON and ECC RET on muscle and tendon tissue in both young and older individuals. We observed that the biomechanical properties of the PT for both YM and OM adapted after only 4 weeks of submaximal RET, with no differences between contraction type. However, we observed that whilst the YM groups appeared to plateau after 4 weeks of submaximal RET, the older groups continued to increase from weeks 4 to 8, suggesting a delayed response. In line with previous research utilising high-intensity RET (~ 80% 1RM), we herein confirm that the distinct contraction-specific muscle architecture remodelling still occurs in response to submaximal RET. This is to say that PA changes were greater following CON RET and Lf changes greater following ECC RET, in both YM and OM. Finally, when considering MTU adaptations as a whole, it is apparent that muscle and tendon adaptations occur synchronously in both YM and OM and irrespective of contraction mode (i.e. CON or ECC), presumably to maintain the efficacy of the MTU. This is in agreement with previous acute studies which observed coordinated muscle and tendon metabolic responses to RET [44].

### The patellar tendon responds equally to submaximal CON and ECC RET

Our data herein demonstrates that the PT of both YM and OM adapts positively to submaximal RET through changes in Young’s modulus and stiffness. This therefore suggests that the strain applied during submaximal RET is sufficient to elicit adaptation, even in an older tendon. However, this is in contrast to a recent paper by Eriksen and colleagues whereby the authors compared moderate-intensity exercise intervention to high-intensity RET over a 12-month period with a similar aged group (~ 67yrs) [17]. In response to the moderate-intensity training, they surprisingly observed that tendon stiffness decreased [17]. Whilst these results are in direct conflict to that herein, it is worth highlighting that Eriksen et al. utilised a resistance band form of training. Thus, the type of loading provided by auxotonic contractions (in which the load increases with the increase in joint extension, such as with resistance bands) is substantially different from isotonic contractions in which the load is approximately constant throughout the joint movement. Hence, a direct comparison of different types of loading (i.e. auxotonic, isometric or isokinetic) may be challenging due to the inherent difference in loading rates that would occur during contraction.

Significantly, we observed no differences in either absolute or percentage change values for Young’s modulus or tendon stiffness at any time between CON and ECC groups for both YM and OM. This lack of difference occurred despite significantly higher training loads in the ECC groups. Indeed, these findings are consistent with previous work completed in YM whereby a higher intensity of RET was used (80% 1RM) [42]. However, our study is the first to compare the effect of isolated CON and ECC RET on tendon mechanical properties in OM. Thus, our data adds further evidence to suggest that the tendon may be “blind” to the direction of strain it is exposed to, in both YM and OM. However, there is evidence to suggest that this may not be the case. Indeed, a recent study by Kubo et al. demonstrated that changes in PT stiffness are greater after CON RET in YM [35]. However, the training loads used by Kubo and colleagues were equal (in absolute terms) for both CON and ECC contractions, implying that the ECC group trained at a lower relative intensity than CON. As aforementioned, tendon adaptation has been shown to be dependent upon the amount of strain [1, 38] and hence training load,it is therefore possible that, for Kubo’s study, CON RET resulted in greater changes due to its higher relative intensity than ECC. Therefore, taken collectively, it is possible that there would be no differences in terms of tendon biomechanical adaptation when CON and ECC components are matched for relative intensity.

### The older tendon still adapts but may require a longer period to respond maximally

In comparison to many previous studies which typically investigated pre- and post- investigation, we utilised multiple time points over a single intervention allowing for a complete understanding of the time course of tendon adaptations. As such, we demonstrate that significant increases in Young’s modulus can occur after only 4 weeks of RET in both YM and OM, irrespective of contraction modality (Fig. 1). However, for YM, the values for Young’s modulus appeared to be approaching a plateau by this time point (i.e. week 4). There are a few possible explanations as to why an apparent adaptation plateau is reached in the YM group, including the moderate load utilised herein. As aforementioned, tendon adaptation is dictated by the acute strain applied to the tendon and hence the training load [1, 38]. Indeed, for YM, significantly larger changes in Young’s modulus have previously been seen with 80% 1RM CON or ECC RET (+ 70% and + 84% respectively) [42]. Thus, is it possible that this plateau may not have been observed if a higher intensity of RET was utilised. Nonetheless, a previous study which investigated Achilles tendon adaptation in response to high load ECC loading in YM also observed a plateau of adaptation after 4 weeks [26]. Therefore, it is possible that even with a higher intensity of training, whilst the magnitude of change may have been larger, this plateau may still have been reached.

In contrast to YM, Young’s modulus continues to increase from weeks 4 to 8 in OM, irrespective of contraction. This therefore suggests that whilst the large majority of tendon adaptations occur within this initial 4-week period for YM, an older tendon requires at least the full 8 weeks. These findings have clear implications for future interventions targeting PT adaptation in OM; such that in response to submaximal RET, the PT of OM requires at least 8 weeks to experience maximal adaptation. There are of course several possible explanations as to why the tendon may adapt slower in OM. One potential avenue of thought is differences in tendon fractional synthesis rates (FSR). However, it has previously been shown that basal FSR rates of tendon tissue are unaffected by ageing [51] and it remains unknown whether acute tendon FSR differs in response to RET between YM and OM. Aside from FSR, it is likely that other age-related changes occur which could impact the rate of tendon adaptation. It has previously been shown that there is an increase in advanced glycation end products within the aged tendon [9, 10]. It has been suggested that this increase in advanced glycation end products can negatively affect collagen fibril sliding [18, 39] and is believed to interfere with protein interactions within the matrix and between the matrix and resident tendon cells [63]. Therefore, it is possible that the increased incidence of AGEs and the consequent interference may, to some extent, impede the ability of the tendon to either sense or transduce mechanical strain, hence slowing adaptation in the elderly.

Despite the possibility that OM require a longer period of time to adapt, a striking finding of this study was that both YM and OM completed the study with very similar values for Young’s modulus (YC, 1.51 GPa vs OC, 1.43GPa; and YE, 1.46 GPa vs OE, 1.34 GPa) (Fig. 1). Taken collectively, these data suggest that the effective restoration of the mechanical properties of an older tendon to that of a younger counter part is likely possible; however, as aforementioned, the time course of PT adaptation in OM to submaximal RET may be slower.

### Distinct architectural remodelling still occurs with submaximal RET and in ageing

In line with previous work which utilised high-intensity RET [2, 23, 24, 57, 67], we show that PA appears to be preferentially increased after CON RET and Lf to undergo greater changes after ECC only RET in YM. Similarly, in OM, these two distinct patterns of architectural remodelling were also seen, demonstrating these changes are not impacted by age. Therefore, these data support the idea that different loading regimes can result in distinct remodelling strategies [19, 22, 49, 66]. Nonetheless, despite the same pattern in both YM and OM, two minor differences between the age groups can be noted. Firstly, the percentage change from baseline in VL Lf appears to occur to a smaller degree in OE compared to the YE over the whole training period (especially at week 2, Fig. 3), perhaps highlighting a slightly dampened capacity for older muscle to adapt. Secondly, significant changes in PA were observed in the OE group as early as 4 weeks of training, whereas PA was only significantly increased at week 8 in YE. This could therefore mean that ECC RET, when performed with moderate load and higher training volume, could also lead to changes in PA as well as Lf, which may be of particular benefit in older individuals.

### Young and older muscle benefit alike to submaximal RET

In both YM and OM, total muscle hypertrophy (i.e. quadriceps muscle volume at week 8) was similar between CON and ECC RET. In YM, there also was no difference in percentage change between the two groups at week 4, suggesting that in response to submaximal RET, CON and RET result in a similar size and speed of adaptation. This observation is in line with previous studies that utilised higher intensities of training, such as 100% MVC [4], 80% of either CON or ECC 1RM [21] or maximum voluntary isokinetic loading within volunteer model (i.e. one limb trained CON and the other trained ECC + CON) [43]. However, in contrast to YM, the relative change in quadriceps volume was significantly greater in OE compared to OC at the 4-week time point (2.8 ± 1.1% vs. 1.3 ± 1.1%, respectively, P < 0.05). This may therefore suggest that for OM ECC-only, moderate load RET could induce greater hypertrophic responses earlier than the CON-only moderate load modality.

Significantly, we observed no differences in the time course or relative (percentage) change in quadriceps muscle volume between respective YM and OM groups. Whilst this may seem surprisingly at first, Kumar and colleagues demonstrated that acute muscle protein synthesis (MPS) rates plateau at 60% 1RM in the older males [36], whilst for younger individuals, this peak occurred at a higher intensity (~ 75% 1RM). Thus, this suggests that whilst training at 60% of 1RM may have been optimal for older males (in an acute MPS sense), it may have been underloaded for the young.

Whilst muscle hypertrophy was observed after only 4 weeks of RET in both YM and OM, increases in isometric MVC were only detected following 8 weeks of training. Significantly, there was no difference between CON and ECC in either YM or OM. It is possible that a large individual variability (YC = 8.3 ± 9%, YE = 12.8 ± 14.1%, OC = 17.8 ± 11.6%, OE = 16 ± 13.1%) may have prevented this from being detected earlier, especially considering that training load significantly increased over time for both ECC and CON RET (Table 3). These significant increases in training load and isometric MVC confirm that moderate load training was successful in providing sufficient stimulus for training load-specific adaptations overtime, as suggested by previous reports [30, 37]. This has significant implications for clinical settings whereby the recovery of muscle mass and strength is essential but heavy load RET is counter advised.

### Angle–torque relation changes in the younger cohort

Whilst peak MVC simply considers the highest force produced at any angle, it is also important to consider the impact of RET upon the angle–torque relation. Interestingly, the angle–torque relation showed significant changes in response to both CON and ECC RET in YM but not in the elderly. Indeed, for YE, larger post-intervention increases in the torque produced at greater joint angles, i.e. shorter quadriceps muscle lengths (Fig. 6). Conversely, the YC group showed only a significant increase in the torque produced at 80 degrees, i.e. longer quadriceps muscle lengths (Fig. 6). Despite these different responses, comparison of the angle–torque slopes did not reveal any significant differences, neither within groups for the pre-to-post changes nor between contraction type at the same time point. Nevertheless, these findings could suggest that distinct remodelling patterns can affect muscle functional properties, at least for the young cohort. The origins of such training-induced shifts of the angle–torque relation may relate to changes in both muscle fascicle length and tendon mechanical properties [61]. However, as fascicle length increase may potentially reflect an addition of sarcomeres in series [5, 7, 75] we expected an opposite effect on the angle–torque relation (YE becoming stronger at longer muscle lengths) than the one observed. This, alongside a lack of association between changes in optimal angle and changes in PT Young’s modulus, makes these findings of difficult interpretation without speculating on potential changes occurred in sarcomere lengths across muscle regions or in joint angle-dependent neural adaptations [40, 46, 47] Further studies should focus on the investigation of the interactions between muscle plasticity and function with contraction-specific stimuli.

### Limitations

This study has some limitations which should be acknowledged. Firstly, we note that changing from bilateral to unilateral RET from week 5 onwards (i.e. weeks 5–8) may have altered the relative load during training. However, training loads from week 5 onwards were based upon unilateral 1RMs; thus, as both groups were assessed in this manner, we believe that whilst the absolute load may have changed, the relative difference between groups remained. Secondly, we acknowledge that the use of both MRI and ultrasonography for the assessment of PT CSA could have caused inaccuracies in these measures across time points, consequently affecting values of PT Young’s modulus [68]. However, we believe this to have had little bearing on our calculations, as supported via the comparison of methodologies at baseline. Finally, we also wish to acknowledge that this study only recruited male participants, and thus the exclusion of females may limit the application of these results.

## Conclusions

In conclusion, our data suggest that the PT responds similarly to moderate load CON and ECC RET, regardless of age. However, whilst the large majority of biomechanical adaptations occur within the first 4 weeks for YM, a full 8 weeks are required by OM to obtain the same benefits. Despite a potentially slower rate of adaptation, our data implies that an older PT retains a remarkable capacity for adaptation since its biomechanical properties can be almost restored to those of their younger counterparts. Submaximal RET resulted in similar muscular benefits for YM and OM alike, with no differences in muscle volume or MVC, irrespective of contraction modality. This further highlights the benefit that moderate load exercise can provide to older populations. Finally, considering the characteristics of ECC loading modality (i.e. greater load imposed at a lower metabolic cost) and the larger early hypertrophic responses, moderate-intensity ECC exercise can be recognised as a very efficient training modality for counteracting the loss of muscle mass in older individuals.

## Declarations

### Disclaimer

The views expressed are those of the authors and not necessarily those of the National Health Service (NHS), the NIHR or the Department of Health and Social Care.

## Abbreviations

1RM: One repetition max
ACSA: Anatomical cross-sectional area
CON: Concentric
CSA: Cross-sectional area
ECC: Eccentric
EMG: Electromyography
FSR: Fractional synthesis rate
LF: Fascicle length
MPS: Muscle protein synthesis
MRI: Magnetic resonance imaging
MTU: Muscle-tendon unit
MVC: Maximal voluntary contraction
OC: Old eccentric
OE: Old eccentric
OM: Older males
PA: Pennation angle
PT: Patellar tendon
RET: Resistance exercise training
US: Ultrasound
VL: Vastus lateralis
YC: Young concentric
YE: Young eccentric
YM: Young males
